# How Can Bee Colony Algorithm Serve Medicine?

**Published:** 2014-07

**Authors:** Zeinab Salehahmadi, Amir Manafi

**Affiliations:** 1 Department of Information Technology, Bushehr University of Medical Sciences, Bushehr, Iran;; 2 Shahid Beheshti University of Medical Sciences, Tehran, Iran

**Keywords:** Bee colony algorithm, Diagnose diseases system, Medicine

## Abstract

Healthcare professionals usually should make complex decisions with far reaching consequences and associated risks in health care fields. As it was demonstrated in other industries, the ability to drill down into pertinent data to explore knowledge behind the data can greatly facilitate superior, informed decisions to ensue the facts. Nature has always inspired researchers to develop models of solving the problems. Bee colony algorithm (BCA), based on the self-organized behavior of social insects is one of the most popular member of the family of population oriented, nature inspired meta-heuristic swarm intelligence method which has been proved its superiority over some other nature inspired algorithms. The objective of this model was to identify valid novel, potentially useful, and understandable correlations and patterns in existing data. This review employs a thematic analysis of online series of academic papers to outline BCA in medical hive, reducing the response and computational time and optimizing the problems. To illustrate the benefits of this model, the cases of disease diagnose system are presented.

## INTRODUCTION

Honey bees are one of the most well studied social insects.^1^ The honey produced by honey bees has been used for wound healing since ancient time.^2^ It was shown to increase the rate of healing in arterial, venous, infected surgical wounds burns, traumatic wounds, lacerations, diabetic and pressure ulcers. But still in clinical practice, antibiotic resistant bacteria are a threat in treatment measures such as methicillin-resistant *Staphylococcus aureus* or *Pseudomonas* and the associated costs are of great importance.^2^^-^^4^ Honey with its antibacterial potential was shown to be effective against 60 species of bacteria and fungi such as yeasts and some species of *Aspergillus* and *Pencillium*.^5^ The antimicrobial activities against common human pathogens using honey was reported as a dressing of infected wounds.^2^^,^^6^^-^^8^ In ophthalmology, topical honey was demonstrated to be effective in bollous keratopathy, scars of herpetic keratitis and treatment of bacterial conjunctivitis.^9^ The therapeutic properties of honey has opened a window on more studies on honey bees.^10^

In the last two decades, the computational researchers were increasingly interested to natural sciences and especially to biology as a source of modeling paradigms. Several research areas were massively influenced by the behavior of various biological entities and phenomena. It gave birth to most of population-based metaheuristics such as Evolutionary Algorithms (EAs), Particle Swarm Optimization (PSO), Bee colony algorithm (BCA), etc. which could be regarded as belonging to the category of intelligent optimization tools used to solve a computational and complex problem in different areas.^11^

Due to its successful implementation, many related works appeared to promote the performance of the standard BCA in the literature to meet up with challenges of recent research problems being encountered. The major advantages of BCA are simplicity, flexibility and robustness, use of fewer control parameters compared to many other search technique, ease of hybridization with other optimization algorithms, ability to handle the objective cost with stochastic nature and ease of implementation with basic mathematical and logical operations. Interestingly, BCA has been tailored successfully to solve a wide variety of discrete and continuous optimization problems so that some other works have modified and hybridized BCA to other algorithms to further enhance the structure of its framework.^1^

The Bees Algorithm is a new population-based search algorithm, first developed in 2005^12^ and later by other researchers independently.^13^ The algorithm mimics the food foraging behavior of swarms of honey bees. In its basic version, the algorithm performs a kind of neighborhood search combined with random search and can be used for optimization problems. Then the fitness of the sites visited by the scout bees are evaluated and bees that have the highest fitness is chosen as “selected bees” and sites visited by them are chosen for neighborhood search.

**Fig. 1:**
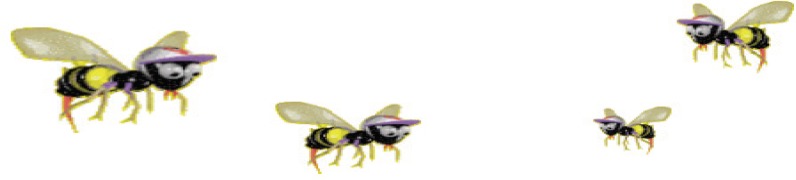
Pseudocode of the basic bees algorithm

Then, the algorithm conducts searches in the neighborhood of the selected sites, assigning more bees to search near to the best sites. Searches in the neighborhood of the best sites are made more detailed by recruiting more bees to follow them than the other selected bees. Together with scouting, this differential recruitment is a key operation of the bees algorithm. The remaining bees in the population are assigned randomly around the search space scouting for new potential solutions. These steps are repeated until a stopping criterion is met. At the end of each iteration; the colony will have two parts, those that were the fittest representatives from a patch and those that have been sent out randomly.The algorithm performs a kind of neighborhood search combined with random search and can be used for both combinatorial and functional optimization (Figure 1).^13^^,^^14^

Pseudocode of the basic Bee Algorithm are as follows: (i) Initializing the population with random solutions; (ii) Evaluating fitness of the population; (iii) While stopping criterion not met, forming a new population; (iv) Selecting sites for neighborhood search; (v) Recruiting bees for selected sites more bees for best sites and evaluating the fitness; (vi) Selecting the fittest bee from each patch; (vii) Assigning the remaining bees to search randomly and evaluate their fitness; and finally (viii) Ending while.^14^

The bee is a social and domestic insect native to Europe and Africa. There are between 60,000 and 80,000 living elements in the hive. The bees feed on nectar as a source of energy in their lives and use pollen as a source of protein in the rearing larvae. Generally, the bee colony contains a single breeding female known as the Queen, a few thousands of males called Drones, a several thousands of sterile females called workers, and any young bee larvae called broods.^14^

The bees share a communication language of extreme precision, based on the dances. These dances are performed by the explorer bee “Scout”. After finding food and returning to the hive, this type of worker “scout” informs others about the distance, direction, quantity and quality of food founded. With their visual, tactile and olfactory perception, the other bees perceive the transmitted information. There are two types of dances; the round dance when food is very close. This dance indicates only the direction. The second type is the waggle dance. It is a dance which forms an eight scheme. It indicates distance and direction of the food source. The distance between the food source and the hive is transmitted depending on the speed of the dance. If dance is faster then, the food distance is smaller. The direction (angle between the food source and the sun relative to the hive) is shown by the inclination of the dance from the vertical with standard deviation of ±3°. The nature of the food is indicated by the odor of the bee when it is rubbed. The amount of food depends on the wriggling of the bee. The more is the wriggling, the more is the quantity.^14^

Bee foraging behaviors are (i) Nest site searching as the most prosperous colonies are reproduced by swarming. In early spring, some queen cells are produced to generate new queen. Before its birth, the old queen leaves the colony with the half of the colony components to form a new colony. They search new nest site. The scouts seek about twelve nest sites. They indicate the various locations of new nests by waggle dances. The dance quality is related to the nest site quality. Thus and over time, selected sites decrease until a single site will be found^14^ and (ii) Food source searching while first some bee “scouts” navigate and explore the region in aim to find a food source. In the positive case, they come at the hive in place called “dance floor” to transmit and share this discovery with the others through dance language as a round or waggle dance relating to the discovery distance. Some bees are recruited and then, become foragers. Their number is proportional to the food quantity information communicated by the scouts. This step is called exploration phase which is followed by the exploitation step. Bee collects food and calculates their quantity to make a new decision. Either it continues collecting by the memorization of this best location, or it leaves the source and returns to hive as simple bee.^14^

There are many potential applications based on the marriage phenomenon among bees which represent the second class of bee colony algorithms. It is based on the mating–flight which can be visualized as a set of transitions in a state–space (the environment) where the queen moves between the different states in the space in some speed and mates with the drone encountered at each state probabilistically. At the start of the flight, the queen is initialized with some energy–content and returns to her nest when the energy is within some threshold from zero or when her spermatheca is full. A drone mates with a queen according to probabilistic factor (the probability of adding the sperm of drone to the spermatheca of queen; that is the probability of a successful mating). The probability of mating is high when either, the queen is still in the start of her mating–flight and therefore her speed is high, or when the fitness of the drone is as good as the queen’s. After each transition in the space, the queen’s speed and energy decay. Honey bee starts with initializing the queen’s genotype at random. After that, the heuristic is used to improve the queen’s genotype, therefore preserving the assumption that a queen is usually a good bee. Afterwards, a set of mating-flights is undertaken. In each mating-flight, the queen’s energy and speed are initialized with some value at random. The queen then moves between different states (i.e. solutions) in the space according to her speed and mates with the drone. If a drone is successfully mated with the queen, his sperm is added to the queen’s spermatheca (i.e. a list of partial solutions). After the queen finishes her mating-flight, it returns to the nest and starts breeding by selecting a sperm from her spermatheca at random followed by crossover with the queen’s genome that complements the chosen sperm. This crossover process results in a brood. This is the haploid–crossover. Mutation then acts on the brood therefore, if the same sperm is used once more to generate a brood, the resultant brood will be different because of mutation. This process is followed by applying the worker to improve the broods. Afterwards, the queen is replaced with the fittest brood if the latter is better than the former. The remaining broods are then killed and a new mating–flight starts.^15^

The Artificial Bee Colony (ABC) algorithm is a swarm based metaheuristic algorithm based on the model first proposed by on the foraging behavior of honey bee colonies.^16^ The model is composed of three important elements including employed and unemployed foragers, and food sources. The employed and unemployed foragers are the first two elements, while the third element is the rich food sources close to their hive. The two leading modes of behavior are also described by the model. These behaviors are necessary for self organization and collective intelligence including recruitment of forager bees to rich food sources, resulting into positive feedback and simultaneously, the abandonment of poor sources by foragers, which causes negative feedback.^17^

In 2005, ABC was developed and evaluated, using multidimensional and multivariable optimization problems. Later on in 2006, the performance of ABC was compared to Genetic Algorithm (GA) on numeric functional optimization, and was later used in 2007 for the training of artificial neural networks. Subsequently in 2007, its performance on different problems was extensively studied and the results were compared with other well known successful algorithms such as GA, PSO, particle swarm inspired evolutionary (PS-EA), differential evolution (DE) and back propagation (BP) algorithms. Similarly in 2008, the performance of ABC was compared with DE, PSO and EA with regards to multidimensional numeric problems. It is important to note that most works were carried out on ABC from 2005 to 2008. In 2009, major studies were undertaken on ABC across different disciplines, where different modifications and hybridizations were attempted by various researchers to tackle various problems in engineering, digital image processing and pattern recognition, protein structure predictions and numerical, real parameter and complex optimization information technology. By 2010, the diversification of ABC into other disciplines was also reported. Prominent among these included scheduling real parameter and other optimization problems, engineering design and information, applied technology and protein structure prediction.^18^

ABC algorithm was used for protein structure prediction using the 3 dimensional hydrophobic-polar model with side-chains 3DHP-SC. Two parallel approaches for the ABC were implemented; a master slave approach and a hybrid-hierarchical approach. Parallel models performance was compared with sequential version for 4 benchmark instances. Results indicated that the parallel models achieved a good level of efficiency and the hybrid hierarchical approach improved the quality of solutions found.^18^

An algorithm was described to be based on combining Fuzzy C mean (FCM) algorithm and ABC algorithm to effectively segment MR images. They calculated the two new parameters; difference between neighboring pixels in the image and the relative location of the neighboring pixels which were introduced by researchers to improve the performance of FCM using the ABC algorithm. Authors compared ABC algorithm with the optimization algorithms such as PSO, GA and ANN and found that the ABC was performed very well in terms of performance speed and not getting entangled in local places. Tremendous increase in the number of ABC publications was witnessed, where series of applications, modifications, parameters tuning and hybridization with different optimization algorithms were used to enhance the performance ABC across various.^19^

A method was used to improve rough set based attribute reduction with ABC algorithm to find the final subset of attributes. The performance was analyzed with five different medical datasets and compared the results with six other reduction algorithms. The result from this approach reached great accuracy of 92.36%, 86.54%, 86.29%, 83.03% and 88.70% respectively.^19^

Automatic Threshold Selection based on ABC algorithm, a new algorithm to select image threshold as a tool to separate objects from the background was automatically based on BCA algorithm. Test results indicated the newly proposed algorithm succeeded in finding optimal threshold to segment images.^19^

An improved algorithm was based on BCA to deal with multi-objective optimization problems. This algorithm was based on the intelligent foraging behavior of honey bee swarm used less control parameters and it was found to be very efficient in solving multimodal and multidimensional optimization problems. The proposed algorithm that used the concept of Pareto dominance to determine the flight direction of a bee and maintained non-dominated solution vectors were found in an external archive. Performance of the proposed algorithm indicated that the proposed method can be considered as a viable alternative to solve multi-objective optimization problems.^19^

In MRI Fuzzy Segmentation of Brain Tissue Proposed algorithm discussed the shortcoming of traditional FCM clustering algorithm for MR images segmentation, which did not guarantee high accuracy especially for noisy or abnormal images. Suggested a modified intensity matrix using ABC before performing FCM clustering algorithm, in order to avoid sensitivity to noise.^19^

In another study on ABC algorithm in optimization feature selection, a new method of feature selection used the ABC algorithm to optimize the selection of features. Experimental results showed that ABC Feature Selection resulted into an optimal feature subset configuration and increased the classification accuracies up to 12% compared to the classifier and standard ensembles.^19^^,^^20^ The main objective of this article was to document developmental knowledge of BCA and its applications. 

## DISCUSSION

In the last few decades, an introduction on several optimization algorithms was developed based on nature-inspired ideas including ant colony optimization, evolutionary algorithm, particle swarm optimization, harmony search, etc. Most of these algorithms were metaheuristic-based searches techniques and generally referred to as multipurpose optimization algorithms, because of their applicability to a wide range of problems as well as in medical domain. A computer assisted detection of exudates in color fundus images of the human retina in diagnosis of retinopathy was previously confirmed. It was proposed that an automatic method would detect hard exudates, a lesion associated with diabetic retinopathy.^21^

Artificial intelligence and machine learning techniques have been used as classification or prediction methods in determining cardiac arrhythmias, coronary artery diseases (CAD) and myocardial infarctions with growing phenomena. CAD is the most common heart disease primarily cause mortality and morbidity in developed or developing countries. The proposed algorithm is repeated 50 times to provide reliability, and variation of the classification accuracy depending on the population size and maximum iteration number which is used as termination criterion for the ABC.^22^

In a survey on the Disease Diagnose System using Machine Learning Algorithm, in the proposed system, the PSO and ABC algorithm gave a better decision, though robust production rules were not known. During preprocessing, the common symptoms were efficiently recognized that converged to best optimal solution. Thus, the implementation of the proposed system gradually reduced the processing time of rules in comparison with the Rule Based System. The algorithm used in the system can be treated as most cases found a solution which represented a good approximation to the optimal one and fast enough for the number of iterations. By the thorough interaction with the users and beneficiaries, the functionality of the system can be extended further to many more areas in and around the world.^23^

However, the attention of researchers in different domains has been drawn to adopt the use of BCA for a variety of decision making problems. This study indicated that BCA approach can predict the risk factors with a relatively high accuracy and provide further supports for the notion that BCA can help medical decision-making process by deciphering the complex interactions between various biological variables and translating the hidden patterns in data into detailed decision-making criteria. BCA is very simple and very flexible when compared to other swarm based algorithms as BCA does not require external parameters such as cross over rate and mutation rate etc., as in case of genetic algorithms, differential evolution and other evolutionary algorithms. The BCA has come to be recognized as a powerful and robust global optimization algorithm, capable of tackling unimodal and multimodal, non-differentiable, nonlinear objective functions. Yet, there still exist many new areas of application and open problems in which BCA could be adopted.^23^

So still BCA remains a promising and interesting algorithm, which would continue to be extensively used by researchers across diverse fields. Its potential advantage of being easily hybridized with different meta heuristic algorithms and components makes it robustly viable for continued utilization for more exploration and enhancement possibilities in many more years to come.

## CONFLICT OF INTEREST

The authors declare no conflict of interest. 

## Footenote

Please cite this paper as:

Salehahmadi Z, Manafi A. How Can Bee Colony Algorithm Serve Medicine?. World J Plast Surg 2014;3(2):87-92.
